# Polycystic ovarian syndrome increases prevalence of concentric hypertrophy in normotensive obese women

**DOI:** 10.1371/journal.pone.0263312

**Published:** 2022-02-25

**Authors:** Kirstie A. De Jong, Filip Berisha, Negar Naderpoor, Alan Appelbe, Mark A. Kotowicz, Kimberly Cukier, Sean L. McGee, Viacheslav O. Nikolaev

**Affiliations:** 1 Institute of Experimental Cardiovascular Research, University Medical Center Hamburg-Eppendorf, Hamburg, Germany; 2 DZHK (German Center for Cardiovascular Research), partner site Hamburg/Kiel/Lübeck, Germany; 3 Metabolic Reprogramming Laboratory, Institute for Mental and Physical Health and Clinical Translation (IMPACT), Metabolic Research Unit, School of Medicine, Deakin University, Waurn Ponds, Victoria, Australia; 4 Department of Cardiology, University Heart and Vascular Center Hamburg, University Medical Center Hamburg-Eppendorf, Hamburg, Germany; 5 Geelong Endocrinology and Diabetes Centre, Geelong, Victoria, Australia; 6 Monash Centre for Health Research and Implementation, Monash University, Clayton, Victoria, Australia; 7 Cardiology Department, Barwon Health, University Hospital Geelong, Geelong, Victoria, Australia; 8 Endocrinology Department, Barwon Health, University Hospital, Geelong, Victoria, Australia; 9 Deakin University, School of Medicine, Waurn Ponds, Victoria, Australia; 10 Department of Medicine-Western Health, Melbourne Medical School, University of Melbourne, Melbourne, VIC, Australia; Zhejiang University College of Life Sciences, CHINA

## Abstract

**Background:**

It remains unclear as to whether polycystic ovary syndrome (PCOS) is an additional risk factor in the development of left ventricular (LV) hypertrophy in obese women. In the current study, we provide clarity on this issue by rigorously analysing patient LV geometry beyond the basic clinical measures currently used. Importantly, the cohort contained only normotensive patients that would normally be deemed low risk with no further intervention required.

**Methods:**

The study comprised 24 obese women with PCOS and 29 obese Control women. Transthoracic echocardiography was used to evaluate LV structure/function. Basic clinical and metabolic data were collected for each participant consisting of age, BMI, blood pressure, fasting glucose, LDL-C, HLD-C, cholesterol and triglyceride levels. Exclusion criteria; BMI < 30 g/m^2^, type 2 diabetes, hypertension.

**Results:**

Both groups exhibited concentric remodelling of the LV posterior wall at a prevalence of ~20%, this associated with grade 1 diastolic dysfunction. Estimated LV mass/height^2.7^ was increased patients with PCOS (45 ± 2.2 vs 37 ± 1.6) with 33% exhibiting LV mass/height^2.7^ above ASE guidelines, compared to 7% in Controls. Furthermore, 25% of patients with PCOS were characterised with concentric hypertrophy, an alteration in LV geometry that was not observed in the Control group.

**Conclusions:**

To our knowledge, this is the first study to assess LV geometric patterns in obese women with PCOS. The results suggest that obese women with PCOS are at greater risk of concentric hypertrophy than obese only women and provide justification for additional cardiovascular risk assessment in normotensive obese/PCOS women.

## Introduction

Polycystic ovarian syndrome (PCOS) is a complex endocrine/metabolic syndrome characterised by the presence of “ovarian cysts”, increased androgens and an irregular or absent menstrual cycle [1]. With common symptoms including impaired fertility, hyperinsulinemia and obesity [2]. There is no cure for PCOS with treatment of the syndrome focused on lifestyle modification, targeted hormonal therapy (androgen blockers) and/or the use of metformin, as such, the syndrome requires long term management. During this time, monitoring of cardiovascular risk is predominately done via assessing co-morbidities that develop as a consequence of PCOS and associated obesity, in particular, the monitoring of associated hypertension. However, this practice assumes that normotensive obese women with PCOS exhibit the same cardiovascular risk as normotensive obese only women.

Previous studies have reported left ventricular (LV) geometric patterns to be predictive of future cardiovascular risk in obese and/or hypertensive patients [3–6]. Indeed, we have previously shown that relative wall thickness (RWT) vs the use of left ventricular posterior wall diameter (LVPWd) is a more sensitive measure to detect LV remodelling in normotensive patients with obesity and/or type 2 diabetes (T2D) and for differing LV geometric patterns to associate with specific grades of diastolic dysfunction (DD) [7], suggesting there is prognostic value in assessing LV geometric patterns in normotensive obese patients. While previous studies have identified increases in left ventricular (LV) mass [8] and increased presence of cardiometabolic risk factors in women with PCOS [9], it is not clear to which extent these factors are driven by the presence of PCOS itself or associated hypertension and/or obesity. Furthermore, while increased LV mass has been reported in PCOS the type of LV remodelling has remained unclear.

In the current study, we provide clarity on this issue by rigorously analysing patient left ventricular structure beyond the basic clinical measures currently used in normotensive obese patients with and without PCOS. This is the first study to our knowledge that has assessed LV geometric patterns in PCOS and provides novel insight into the additive effects of PCOS in LV remodelling in obesity.

## Methods

### Study approval

This multisite study was performed in a cross sectional and retrospective format, A and comprised a total of 23 Control normotensive obese women without PCOS and 21 normotensive obese women with PCOS from the University Hospital Geelong and the Geelong Endocrinology and Diabetes Centre in Australia, as well as a further 6 Control normotensive obese women without PCOS and 3 normotensive obese women with PCOS from the University Heart and Vascular Center Hamburg, Germant. For data collection within the Australian sites the study was conducted in accordance with National Health and Medical Research Council (NHMRC) guidelines and was approved by the Human Research Ethics Committee (HREC) via the Barwon Health Research and Integrity Unit, in accordance to guidelines outlined in Section 5 of the National Statement on Ethical Conduct in Human Research. According to the declaration of Helsinki Hamburg data were obtained retrospectively from patients who underwent echocardiography at the University Heart and Vascular Center Hamburg in the years 2020–2021, all data underwent pseudonymisation prior to data collection and analysis.

### Patient characteristics/exclusion and inclusion criteria

Basic clinical and metabolic data were collected for each patient consisting of age, height, weight, blood pressure, fasting glucose, total cholesterol, LDL-C, HLD-C and triglyceride levels (after an overnight, 8 hour minimum fast). Exclusion criteria included; BMI < 30 g/m^2^, type 2 diabetes, history of hypertension or use of anti-hypertensive medication and history of cardiac disease or systemic disease. PCOS diagnosis was based on the Rotterdam criteria in which two of the following three criteria were present; 1. Oligoovulation or anovulation, 2. Hyperandrogenism, either clinical presentation or biochemical and/or 3. Polycystic ovaries detected via transvaginal ultrasound.

### Blood pressure measurements

Blood pressure (BP) was recorded as per the American Heart Association guidelines, with the use of OMRON *Intelli* sense HEM-907 or HBF-1300. As all participants were obese, it is important to note that an appropriate sized cuff bladder was used, at least 80% of the patient’s arm circumference. In the incidence of an elevated BP reading (≥ 140/90 mmHg), the measurement was repeated up to three times with the lowest measurement recorded.

### Transthoracic echocardiography

All echocardiograms were performed using the Phillips Ie33 with a S5-1 transducer. A combination of two-dimensional, M-mode, pulsed wave and continuous wave Doppler and tissue Doppler were used. Left ventricular diameter and wall thicknesses were measured in the parasternal long axis view using two-dimensional or M-mode measurements; left ventricular internal diameter during diastole and systole (LVIDd, LVIDs), interventricular septum diameter during diastole and systole (IVSd, IVSs), left ventricular posterior wall diameter during diastole and systole (LVPWd, LVPWs). Mitral inflow velocities (E’ velocity, Peak E-wave, Peak A-Wave) and deceleration times (DT) were measured using pulsed wave Doppler in the apical 4 chamber view. Echocardiographic data was analysed using proprietary software.

### Characterisation of diastolic dysfunction

DD was characterised according to ASE guidelines and as previously described [10]. An E’ ≥ 10cm/s was categorised as normal diastolic function. Grade 1 DD (impaired relaxation) was characterised by a decreased E’ < 10cm/s with an E/A < 0.8 and E/E’ ≤ 8. Grade 2 DD (pseudonormal) was characterised by a decreased E’ < 10cm/s with an E/A 0.8–1.5 and E/E’ 9–14. And grade 3 DD (restrictive) was characterised by a decreased E’ < 10cm/s with an E/A ≥ 2 and E/E’ > 14.

### Left ventricular geometry

LV mass was estimated according ASE guidelines as previously described [11], in which LV mass (grams) = (0.8 ∙ [1.04 ∙ (LVIDd + IVSd + LVPWd)^3^ - (LVIDd)^3^]) + 0.6). LV mass was then indexed to body surface area (BSA, g/m^2^) and to height (g/m^2.7^). RWT was calculated using the formula, RWT = 2 ∙ (LVPWd)/LVIDd. LV geometry was characterised using the following criteria; Normal LV geometry, RWT ≤ 42, LVMI (g/m^2.7^) ≤ 51; eccentric hypertrophy, RWT ≤ 42, LVMI (g/m^2.7^) > 51; concentric remodelling, RWT > 42, LVMI (g/m^2.7^) ≤ 51 and concentric hypertrophy, RWT > 42, LVMI (g/m^2.7^) > 51.

### Statistical analysis

Continuous variables were represented as means ± 1 SEM, unless otherwise stated. Means of continuous variables were analyzed via a student’s t test. Associations were determined by performing linear regression analysis, assessed with Pearson’s correlation coefficient. Categorical variables were expressed as percentages or prevalence and analysed via chi-square tests, using Fisher’s exact test. To determine independent predictors of categorical variables, multivariable logistic regression analysis was performed. *p*<0.05 was considered significant. All statistical analysis were performed using SPSS version 23.

## Results

### Basic clinical data

As per selection criteria, women of both groups were of similar age and BMI and all women were normotensive with no differences between systolic and diastolic blood pressure (Table 1). In addition, there were no differences in fasting glucose, total cholesterol, HDL-C, LDL-C and triglycerides. History of metformin use and heart rate were increased in the PCOS group (26.09% vs 42.86% and 71.52 ± 2.50 vs 80.10 ± 3.16 bpm respectively, p <0.05, Table 1).

**Table 1:** Basic clinical data for Control and PCOS groups.

Variable	Control	PCOS	p value
N =	29	24	
Age (years)	38.43 ± 1.34	39.35 ± 2.06	0.812
Height (cm)	165.47 ± 1.51	161.00 ± 2.03	0.396
BMI (g/m^2^)	35.56 ± 1.52	36.23 ± 1.78	0.858
BSA (m^2^)	2.10 ± 0.06	2.08 ± 0.07	0.611
Systolic BP (mmHg)	126.17 ± 2.12	127.10 ± 2.39	0.782
Diastolic BP (mmHg)	78.09 ± 1.98	80.48 ± 1.26	0.335
Pulse pressure (mmHg)	48.09 ± 2.37	46.62 ± 2.35	0.673
Heart rate (bmp)	71.52 ± 2.50	80.10 ± 3.16*	0.046
Glucose (mmol/l)	5.21 ± 0.09	5.40 ± 0.16	0.339
Cholesterol (mmol/l)	5.38 ± 0.16	5.30 ± 0.20	0.949
HDL-C (mmol/l)	1.33 ± 0.06	1.38 ± 0.05	0.546
LDL-C (mmol/l)	2.44 ± 0.14	2.73 ± 0.17	0.219
Triglycerides (mmol/l)	1.58 ± 0.12	1.55 ± 0.13	0.849
Biguanides (%)	26.09	42.86*	0.048

### Patients exhibited normal associations between age and indices of LVH and diastolic function

To increase confidence that our dataset was representative of the wider population, we first confirmed that the dataset exhibited normal associations between age and indices of diastolic decline and left ventricular hypertrophy (LVH). In which bivariate correlation analysis confirmed age to associate with the diastolic indices Peak A wave (p = 0.001) and E/A ratio (p = 0.002) and with the LVH indices; LV mass/BSA (p = 0.048), IVSd (p = 0.044), LVPWd (p = 0.035) and RWT (p = 0.045) (Table 2).

**Table 2:** Associations between age with indices of left ventricular hypertrophy and diastolic function.

Variable	p value
Association of age with indices of left ventricular hypertrophy	
IVSd	0.044*
LVPWd	0.035*
LV mass	0.060
LV mass/BSA	0.048
LV mass/height	0.188
RWT	0.045
*Association of age with indices of diastolic function*	
Peak E Wave	0.399
Peak A Wave	0.001*
E/A ratio	0.002*
E’	0.065
E/E’	0.085
DT	0.444

### Obese women with PCOS exhibited increased LV mass, RWT and prevalence of concentric hypertrophy

**Fig 1:**
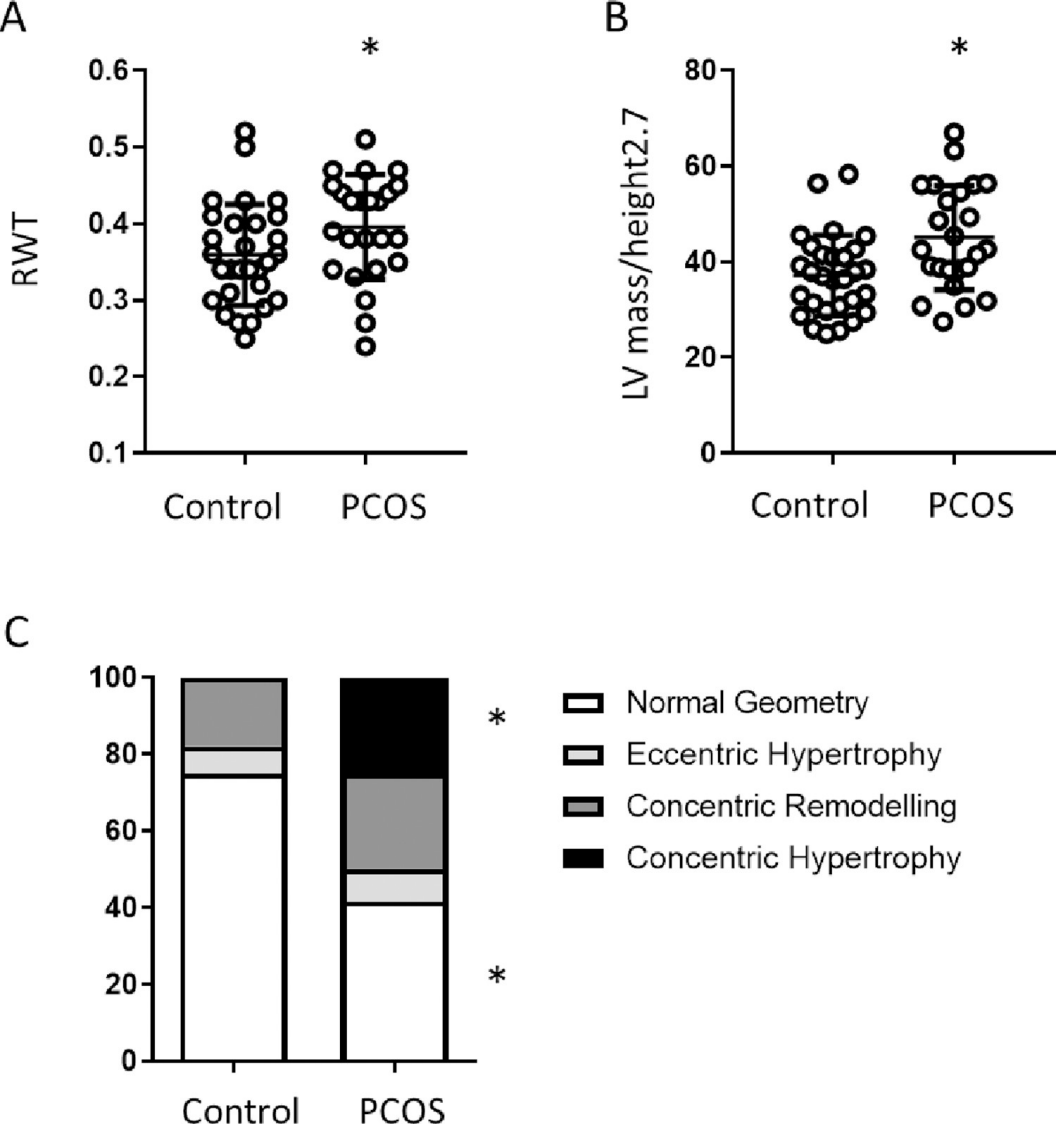
**A.** Relative wall thickness (RWT), **B.** LV mass/height and **C.** Characterisation of left ventricular geometric patterns in Control and PCOS groups, data presented as prevalence. *p<0.05, **p<0.01.

Considering clinical indices of LVH first, comparing obese only to obese women with PCOS, PCOS women exhibited increased LV mass, whether unadjusted (142.16 ± 5.67 vs 161.69 ± 7.69, p<0.05) or normalised to BSA (67.41 ± 2.46 vs 78.53 ± 3.45, p<0.01) or height^2.7^ (45.07 ± 2.17 vs 37.15 ± 1.54 p<0.05) (Table 3. Fig 1B). Furthermore, within the groups, women with PCOS showed a greater prevalence of LV mass above ASE guidelines (33% vs 7% respectively (p<0.05)). To gain a greater understanding of the type of LV remodelling observed, LV geometric patterns were assessed. In which LV mass/height^2.7^ measurements were utilised with the additional measure of RWT (Fig 1A–1C). Comparing Control and PCOS groups, the prevalence of women with normal LV geometry was reduced in the PCOS group (75% vs 41.67% p<0.05), while the prevalence of eccentric hypertrophy (7.14% vs 8.33%) and concentric remodelling (17.86% vs 25%) were comparable between both groups. However interestingly, women with PCOS exhibited a 25% prevalence of concentric hypertrophy, compared to 0% in the obese only group.

**Table 3:** Echocardiography data for Control and PCOS groups.

Variable	Control	PCOS	p value
Aortic root (cm)	3.00 ± 0.07	2.94 ± 0.07	0.617
Ascending aorta (cm)	3.72 ± 0.16	3.13 ± 0.13	0.162
LAVi (mL/m^2^)	28.44 ± 1.65	29.57 ± 2.56	0.787
IVSd (cm)	0.85 ± 0.02	0.98 ± 0.04*	0.014
LVIDd (cm)	4.86 ± 0.08	4.80 ± 0.09	0.624
LVIDs (cm)	3.33 ± 0.23	3.12 ± 0.12	0.545
LVPWd (cm)	0.86 ± 0.02	0.94 ± 0.03*	0.032
LV mass (g)	142.16 ± 5.67	161.69 ± 7.69*	0.051
LV mass/BSA	67.41 ± 2.46	78.53 ± 3.44*	0.013
Peak E-Wave	0.91 ± 0.03	0.93 ± 0.03	0.787
Peak A-Wave	0.64 ± 0.03	0.58 ± 0.03	0.321
E/A Ratio	1.50 ± 0.07	1.64 ± 0.13	0.435
E’	9.5 ± 0.53	9.95 ± 0.37	0.526
E/E’	10.19 ± 0.52	10.16 ± 0.66	0.807
DT	221.73 ± 20.12	210.94 ± 18.41	0.753
EF (%)	60.60 ± 2.42	62.70 ± 3.54	0.699
FS (%)	33.18 ± 1.53	35.41 ± 2.46	0.543
SV	74.98 ± 3.65	75.07 ± 3.97	0.504
CO	5.47 ± 0.31	5.92 ± 0.36	0.442

### Diastolic function was comparable between obese women without and with PCOS

**Fig 2:**
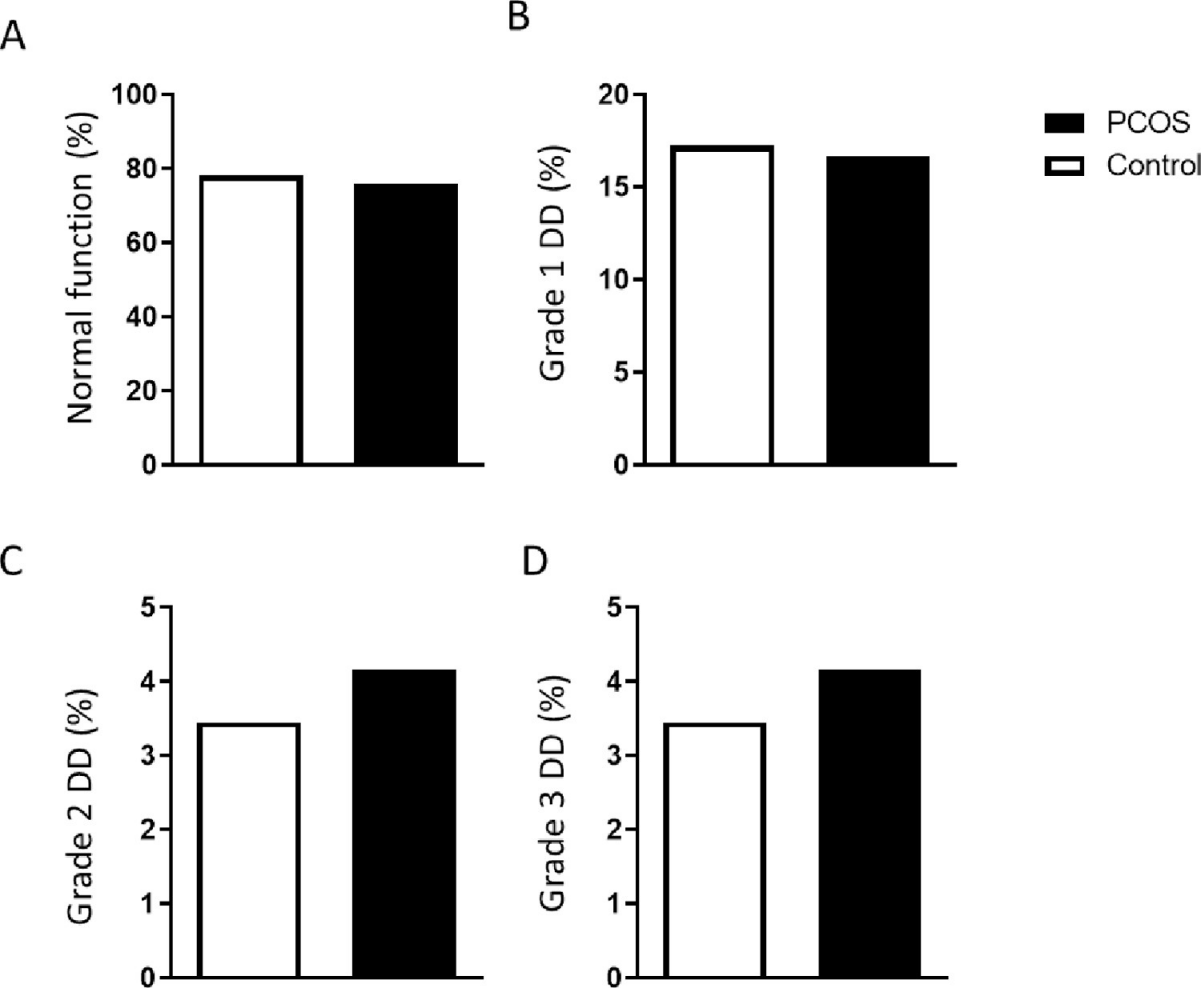
Diastolic function characterisation in Control and PCOS groups, **A.** Normal diastolic function, **B.** Grade 1 diastolic dysfunction**, C.** Grade 2 diastolic dysfunction and **D.** Grade 3 diastolic dysfunction, data presented as prevalence within each group.

Diastolic function was comparable amongst obese women without and with PCOS, with both groups exhibiting a prevalence of normal diastolic function of ~70%, as well as a comparable prevalence of Grade 1 DD (17.24% vs 16.67%) and Grade 2 and 3 DD (3.45% vs 4.17%) (Fig 2A–2D). Bivariate correlation analysis identified concentric remodelling to associate with grade 1 DD (r = 0.49, p = 0.002), when controlling for age and BMI this association was attenuated, however still significant (p = 0.048). Interestingly, concentric hypertrophy did not associate with diastolic dysfunction as we have previously observed [7].

## Discussion

The results from the current study provide novel insights into alterations in LV geometric patterns in obese women with PCOS. The major findings from the current study are the identification of concentric hypertrophy in the PCOS group, that was not accompanied by the presence of DD, suggesting that the PCOS heart has undergone compensatory alterations in LV structure that are not present in obese only women.

Clinically, LVH is commonly assessed via transthoracic echocardiography in which 2 dimensional and M-Mode measurements are used to determine the LVPWd, LVIDd and estimated LV mass [11]. More commonly used in research settings is the characterisation of LV geometric patterns, for which there are four types; normal, eccentric hypertrophy (increase in LV mass), concentric remodelling (increase in the RWT) and concentric hypertrophy (increase in both LV mass and RWT) [11]. The advantage of determining LV geometric patterns is that it requires the additional assessment of the RWT, which is the thickness of the LVPW in relation to the other dimensions of that individual’s heart. Therefore, this value is not reliant on standard normal reference ranges, which vary with age, gender and ethnicity [12]. We have previously shown that RWT vs the use of LVPWd is a more sensitive measure to detect LV remodelling in normotensive obese and/or T2D patients and for differing LV geometric patterns to associate with specific grades of DD [7]. Consistent with these past observations, in the current study the presence of concentric remodelling associated with grade 1 DD. However, in contrast, in the current study the presence of concentric hypertrophy was not accompanied by grade 3 DD. The main difference between the two studies in question is age, with the women in the current studying averaging an age of ~20 years younger. It is therefore tempting to speculate that the presence of concentric hypertrophy in the current study may be a compensatory response to PCOS and an early indicator of future diastolic dysfunction risk in these women.

It is difficult to pin point the exact mechanism/s involved in the pathogenesis of the development of concentric hypertrophy in these PCOS women. This in part due to the complex and multifaceted nature of PCOS and its associated syndromes, such as hyperlipidaemia, hypertension, insulin resistance and hyperandrogenism, thus making it difficult to dissect the individual contributions that these cardiovascular risk factors may have as well as their interacting influences in promoting LV remodelling. Interestingly, while the main cardiovascular preventative strategy in PCOS focuses on treating these commonly associated cardiovascular risk factors, the patients in the current study exhibited normal fasting glucose and lipid profiles (possibly in part, accounted by the increased use of metformin), yet the PCOS group still presented with a type of LV remodelling not observed in the obese only group suggesting that normalising some of the associated metabolic abnormalities in PCOS may be insufficient to mitigate increased cardiovascular risk. Importantly, essential data such as fasting plasma insulin and androgen levels were missing from our study. Indeed the lack of this data reflects common clinical practice in the monitoring and treatment of PCOS in which plasma insulin levels are rarely assessed and androgen levels are assessed at the time of diagnosis and will only be reassessed if androgen blocking therapy is required. It is therefore challenging to understand the associations between insulin resistance and androgen levels with LV remodelling in PCOS patients retrospectively in a clinical setting. However, rat models with PCOS like phenotypes have helped to shed some light into the influence of these factors in LV remodelling, with testosterone treatment shown to induce LV hypertrophy with reports of increased LV mass [13], LVPWd and cardiomyocyte cross sectional area [14]. While insulin resistance is known to be a strong promoter of hypertrophy in obesity and type 2 diabetes [15] the effects of insulin resistance in promoting LV remodelling specifically in PCOS is less clear. Moving forward, long term prospective studies in which echocardiography and comprehensive androgen and metabolic blood profiling of women with PCOS at time of diagnosis and in the years proceeding will be imperative to understand which of these factors may be driving LV remodelling in PCOS.

The strengths of the current study included the inclusion of only obese and normotensive patients, thereby allowing us to tease out the additive effects of PCOS in obesity, independent of hypertension. In addition, the use of transthoracic echocardiography and its application to assess LV geometric patterns is another strength of the study as it maximises translational potential of the study, opposed to assessment of cardiac structure and function by less accessible and more intrusive methods such as Magnetic Resonance Imaging and catheterization. The main limitation however of the current study was our relatively low sample numbers, therefore in order to build confidence that the dataset were representative of the wider population we confirmed that the patients exhibited normal associations between age with diastolic decline and LV structure, thereby allowing us to exclude the presence of “outliers” that skewed the results of the study. Furthermore, the study was conducted with data obtained from multiple sites within Australia and Germany allowing us to confirm associations and LV remodelling patterns in different subpopulations thereby further strengthening the confidence in the results obtained.

In summary this study has identified the presence of concentric hypertrophy in normotensive obese PCOS patients that was not observed in normotensive obese only patients, suggesting that PCOS is an independent risk factor for LV remodelling in obesity. Therefore, this study provides justification for additional cardiovascular risk assessment in normotensive obese PCOS patients.

## Supporting information

S1 File (PDF)

## Abbreviations

ASE: American Society of Echocardiography
BMI: Body mass index
BP: Blood pressure
BSA: Body surface area
CO: Cardiac output
DD: Diastolic dysfunction
DT: Deceleration time
EF: Ejection fraction
FS: Fractional shortening
HLD-C: High density lipoprotein cholesterol
IVS: Interventricular septum
LDL-C: Low density lipoprotein cholesterol
LVH: Left ventricular hypertrophy
LVID: Left ventricular internal diameter
LVPW: Left ventricular posterior wall
PCOS: Polycystic ovarian syndrome
RWT: Relative wall thickness
SV: Stroke volume
T2D: Type 2 diabetes
