# Supplementary material for: Polycystic ovarian syndrome increases prevalence of concentric hypertrophy in normotensive obese women
Source: PLoS One. 2022 Feb 25;17(2):e0263312. doi: 10.1371/journal.pone.0263312 (PMC8880941; doi:10.1371/journal.pone.0263312)
Supplement: S1 File — (PDF) [file pone.0263312.s001.pdf]

Table 1. M-Mode

PCOS	Aortic Root (cm)	Left Atrium (cm)	LA Vol index (mL/m^2)	LV diastolic (cm)	LV systolic (cm)	IV Septum (cm)	Posterior Wall (cm)
0	3,1		30	5,2	3,4	0,7	0,7
0	2,6			4,7		0,7	0,7
0	2,9		48	5,3	3,4	0,8	0,9
0	2,7			5,3	0,29	1	0,9
0	2,8		40	5,1	3,4	0,7	0,7
0	3,1			4,7	3,1	0,9	1
0	2,4	2,1	19	4,1	2,8	0,8	0,7
0	2,9		22	4,2	2,8	0,9	1,1
0	3,1			5	3	0,9	0,8
0	3,6		37	5,3		1	0,9
0	3,1	3,7	19	5,4	3,9	0,8	0,8
0	2,2		25	4,7		0,8	1
0	2,6		31	5	3,1	0,6	0,7
0	3,3	3,3	36	4,7	2,8	0,9	0,9
0	3,2	2,9	34	4,7		0,9	0,8
0	3,8	3,8		5,6	3,3	0,8	1
0	3,1	5,2		5,2	6,2	0,6	0,8
0	2,6	3,2	32	4,8	3,3	0,7	0,6
0	2,9		16	4,2	2,7	1	0,9
0	3,1	5,2		5,6	6,2	0,6	0,8
0	3,1	4,1	22	5,2	3,3	0,9	0,9
0	3,3		27	4,9	2,9	1	1
0	3,6		17	4		1,1	1
0				4,9		1	0,9
0				5,3		1	1
0				4,4		0,8	0,8
0				4,5		0,9	0,9
0				4		0,8	0,8
0				4,9		1	1
1	2,9	2,5	23	4,2	2,5	1	0,9
1		3,8	14	4,7	3,3	1,2	1,1
1	3,1	3,9		4,7	2,1	1,1	1,1
1	3,1		32	4,1	3,3	1	0,9
1		3,8	14	4,7	3,3	1,2	1,1
1	2,7	2,9	26	5,8	3,6	0,7	0,7
1	3,1		30	5,2	3,4	0,7	0,7
1	2,6			4,7		0,8	0,9
1	2,7			5,3	2,9	1	0,9
1				4,7	2,4	0,9	0,9
1	2,4	2,1	19	4,1	2,8	1,1	0,9

1	3,3			4,7	2,5	1,1	1
1	3,1			4,7	2,4	1	1
1	3,2	2,5	31	4,9	3,9	0,7	0,8
1	2,2		25	4,7		0,8	1
1	2,8		38	4,6	4,3	0,8	0,8
1	2,8	2,7	30	5,3	4	0,7	0,8
1	3,1		67	5,1	3,4	1,1	1
1	3,8	3,8		5,8	3,3	0,8	1
1	3,3	3,3	36	4,7	2,8	0,9	0,9
1	2,8		29	4,7		1,6	1,2
1				4,85		1,2	1,1
1				4,85		1,1	0,93
1				4		1	0,9

0 = non PCOS, 1 = PCOS

Table 2. M-Mode Calculations

PCOS	LV MASS	LVEDV	LVESV	EF	FS	BSA	LV Mass/BSA	RWT	LV Mass/height ^2.7
0	122,8108	129,5074	47,43586	63,37207	34,61538	2,0011	61,37165	0,269231	30,75404
0	103,0675	102,3607				1,8343	56,18899	0,297872	33,27698
0	162,1103	135,3427	47,43586	64,9513	35,84906	1,6959	95,58956	0,339623	58,3523
0	187,2767	135,3427				2,2307	83,95422	0,339623	45,41398
0	118,7224	123,8076				2,1335	55,64676	0,27451	27,45401
0	153,4159	102,3607	37,91582	62,95862	34,04255	1,9755	77,65929	0,425532	38,41811
0	89,37024	74,22262	29,55077	60,1863	31,70732	1,7551	50,92031	0,341463	25,99075
0	137,2477	78,57818	29,55077	62,39316	33,33333	1,7475	78,53944	0,52381	37,93806
0	146,8348	118,2432	35	70,4	40	1,8791	78,14103	0,32	36,18212
0	187,2767	135,3427				2,7351	68,4716	0,339623	43,30688
0	154,966	141,3138	65,91	53,35914	27,77778	2,4603	62,98661	0,296296	36,40381
0	142,7073	102,3607				1,5759	90,55604	0,425532	41,50228
0	104,6391	118,2432	37,91582	67,93405	38	1,7671	59,21516	0,28	29,41508
0	142,7073	102,3607	29,55077	71,13075	40,42553	2,1907	65,14231	0,382979	56,43577
0	132,3231	102,3607				2,0259	65,3157	0,340426	32,08791
0	191,6339	166,559	44,13316	73,50299	43,10345	2,1795	87,9256	0,357143	46,47058
0	122,8108	272,16	193,9879	28,72284	13,88889	2,1795	56,34816	0,307692	29,78122
0	97,43565	107,52	44,13316	58,95354	31,25	1,8039	54,01389	0,25	25,62356
0	127,807	78,57818	27,01588	65,6191	35,71429	1,8867	67,74102	0,428571	33,06343
0	139,8635	272,16	193,9879	28,72284	13,88889	2,3375	59,83465	0,285714	31,34883
0	168,9901	129,5074	44,13316	65,92228	36,53846	2,1795	77,5362	0,346154	40,97956
0	176,0355	112,8141	32,21189	71,44693	40,81633	2,2819	77,14427	0,408163	42,68804
0	136,2002	70				1,7663	77,11045	0,5	37,02433

0	164,3235					2,4031	68,37978	0,367347	39,21818
0	200,3965					2,8983	69,14277	0,377358	40,98777
0	109,4389					2,3671	46,23333	0,363636	28,78017
0	132,8231					2,0811	63,82351	0,4	37,97532
0	93,46451					2,5147	37,16726	0,4	24,9885
0	176,0355					2,8083	62,68402	0,408163	45,54007
1	127,807	78,57818	22,32143	71,59335	40,47619	1,8891	67,65496	0,428571	67,03236
1	199,5953	102,3607	44,13316	56,88467	29,78723	2,4071	82,91939	0,468085	56,10819
1	187,5388	102,3607	14,406	85,92624	55,31915	2,4071	77,91066	0,468085	52,71899
1	122,9697	74,22262	44,13316	40,53947	19,5122	1,7075	72,01741	0,439024	31,81204
1	199,5953	102,3607	44,13316	56,88467	29,78723	2,4071	82,91939	0,468085	56,10819
1	148,8092	166,559	54,432	67,31969	37,93103	1,7527	84,90281	0,241379	39,13373
1	122,8108	129,5074	47,43586	63,37207	34,61538	2,0011	61,37165	0,269231	30,75404
1	132,3231	102,3607				1,8343	72,13818	0,382979	42,72262
1	187,2767	135,3427	32,21189	76,19977	45,28302	2,2307	83,95422	0,339623	45,41398
1	142,7073	102,3607	20,16	80,30494	48,93617	1,7407	81,98269	0,382979	38,79319
1	132,1059	74,22262	29,55077	60,1863	31,70732	1,7551	75,26974	0,439024	38,41918
1	175,8267	102,3607	22,32143	78,19336	46,80851	2,9123	60,37382	0,425532	48,60209
1	164,4541	102,3607	20,16	80,30494	48,93617	2,2451	73,25022	0,425532	42,54397
1	120,8198	112,8141	65,91	41,57646	20,40816	2,6131	46,23621	0,326531	27,50513
1	142,7073	102,3607				1,5759	90,55604	0,425532	41,50228
1	117,9053	97,336	83,06701	14,65951	6,521739	1,9123	61,6563	0,347826	30,5019
1	138,3418	135,3427	70	48,27945	24,5283	1,8055	76,62241	0,301887	38,88923
1	200,7767	123,8076	47,43586	61,68582	33,33333	1,8303	109,6961	0,392157	54,57865
1	203,4948	166,559	44,13316	73,50299	43,10345	2,1795	93,36768	0,344828	49,34684

1	142,7073	102,3607	29,55077	71,13075	40,42553	2,1907	65,14231	0,382979	56,43577
1	265,2193	102,3607				2,3263	114,0091	0,510638	63,29844
1	209,7995	110,1502				1,7979	116,6914	0,453608	56,09162
1	176,6317	110,1502				2,5031	70,56517	0,383505	38,38702
1	118,2273	70				1,8643	63,41647	0,45	34,9775

0 = non PCOS, 1 = PCOS

Table 3. LV geometric patterns

PCOS	Normal LV geometry	Eccentric Hypertrophy	Concentric Remodelling	Concentric hypertrophy
0	1	0	0	0
0	1	0	0	0
0	0	1	0	0
0	1	0	0	0
0	1	0	0	0
0	0	0	1	0
0	1	0	0	0
0	0	0	1	0
0	1	0	0	0
0	1	0	0	0
0	1	0	0	0
0	0	0	1	0
0	1	0	0	0
0	0	1	0	0
0	1	0	0	0
0	1	0	0	0
0	1	0	0	0
0	1	0	0	0
0	0	0	1	0
0	1	0	0	0
0	1	0	0	0
0	1	0	0	0
0	0	0	1	0
0	1	0	0	0
0	1	0	0	0
0	1	0	0	0
0	1	0	0	0
0	1	0	0	0
0	1	0	0	0
1	0	0	0	1
1	0	0	0	1
1	0	0	0	1
1	0	0	1	0
1	0	0	0	1
1	1	0	0	0
1	1	0	0	0
1	1	0	0	0
1	1	0	0	0
1	1	0	0	0
1	1	0	0	0

1	0	0	1	0
1	0	0	1	0
1	1	0	0	0
1	0	0	1	0
1	1	0	0	0
1	1	0	0	0
1	0	1	0	0
1	1	0	0	0
1	0	1	0	0
1	0	0	0	1
1	0	0	0	1
1	1	0	0	0
1	0	0	1	0

0 = No PCOS, 1 = PCOS. For LV geometric patterns 0 = no presence, 1 = presence

Table 4. Diastolic function

PCOS	Normal Diastolic function	Grade 1 DD	Grade 2 DD	Grade 3 DD
0	0	0	1	0
0	1	0	0	0
0	1	0	0	0
0	1	0	0	0
0	1	0	0	0
0	0	1	0	0
0	1	0	0	0
0	1	0	0	0
0	1	0	0	0
0	0	0	1	0
0	0	0	1	0
0	1	0	0	0
0	1	0	0	0
0	1	0	0	0
0	1	0	0	0
0	0	0	1	0
0	1	0	0	0
0	1	0	0	0
0	0	0	1	0
0	1	0	0	0
0	1	0	0	0
0	1	0	0	0
0	0	0	0	1
0	1	0	0	0
0	1	0	0	0
0	1	0	0	0
0	1	0	0	0
0	1	0	0	0
0	1	0	0	0
1	1	0	0	0
1	1	0	0	0
1	1	0	0	0
1	1	0	0	0
1	1	0	0	0
1	1	0	0	0
1	1	0	0	0
1	1	0	0	0
1	0	0	1	0
1	1	0	0	0
1	1	0	0	0

1	0	0	0	1
1	0	0	1	0
1	1	0	0	0
1	1	0	0	0
1	0	0	1	0
1	1	0	0	0
1	1	0	0	0
1	0	0	1	0
1	0	1	0	0
1	1	0	0	0
1	1	0	0	0
1	1	0	0	0
1	1	0	0	0

0 = No PCOS, 1 = PCOS. For diastolic function 0 = no presence, 1 = presence
